# Impact of the introduction of a nucleic acid amplification test for *Clostridium difficile* diagnosis on stool rejection policies

**DOI:** 10.1186/s13099-018-0245-x

**Published:** 2018-05-30

**Authors:** J. Goret, J. Blanchi, P. Floch, O. Peuchant, D. Chrisment, R. Sanchez, H. Biessy, R. Lemarié, D. Leyssene, B. Loutfi, S. Mimouni, T. Flao, C. Bébéar, F. Mégraud

**Affiliations:** 1 Laboratoire de Bactériologie, C.H.U. de Bordeaux, Groupe Hospitalier Pellegrin, Place Amélie Raba Léon, 33076 Bordeaux Cedex, France; 2 C.H.U. de Bordeaux, Hôpital Haut-Lévèque, Pessac, France; 3 C.H. de Dax-Côte d’Argent, Dax, France; 4 C.H. de Périgueux, Périgueux, France; 5 G.H. de La Rochelle-Ré-Aunis, La Rochelle, France; 6 C. H. de la Côte Basque, Bayonne, France; 7 C.H. Mont de Marsan, Mont de Marsan, France; 8 C. H. Arcachon, Arcachon, France; 9 C.H.I.C Marmande-Tonneins, Marmande, France

**Keywords:** *C. difficile*, Diagnosis, Impact, Molecular, Algorithm, NAAT

## Abstract

**Background:**

The change from non-molecular to nucleic acid amplification tests (NAATs) is known to increase the detection of *Clostridium difficile* infection (CDI); however, the impact on stool rejection policies in clinical laboratories is unclear. The current guidelines have reinforced the importance of respecting strict conditions for performing tests on stool samples for CDI diagnosis. The purpose of this study was to estimate whether the implementation of molecular tests has resulted in changes in stool rejection policies between clinical laboratories that introduced NAATs and those that did not.

**Results:**

A survey was conducted to evaluate the change in the number of stool samples rejected and the rejection criteria among 12 hospital laboratories in southwestern France before and after the switch from non-molecular tests to NAATs using retrospective data from June 1 till September 30, 2013 and the same period 2014. Four laboratories introduced NAATs as a second or third step in the process. A total of 1378 and 1297 stools samples were collected in 2013 and 2014, respectively. The mean number of rejected stool samples significantly increased (p < 0.001, Chi square test), with a total of 99 (7.1%) and 147 (11.3%) specimens rejected in 2013 and 2014, respectively. Notably, these laboratories had more stringent criteria and were no longer testing the stool samples of patients with CDI-positive results within 7 days. In contrast, there was a significant decrease in the rate of rejected stool samples (p < 0.001, Chi square test) in the five laboratories that did not adopt NAATs and a less stringent stool rejection policy.

**Conclusion:**

Nucleic acid amplification test implementation improved compliance with recommended stool rejection policies. Laboratories should follow the recommended laboratory algorithm for the CDI diagnosis combined with the correct stool rejection policy.

**Electronic supplementary material:**

The online version of this article (10.1186/s13099-018-0245-x) contains supplementary material, which is available to authorized users.

## Background

*Clostridium difficile* infection (CDI) is currently the major cause of healthcare-associated diarrhea. Multiple diagnostic approaches are available for CDI. No single test is suitable for use as a stand-alone test, and current Infectious Disease Society of America (IDSA) and European Society of Clinical Microbiology and Infectious Diseases (ESCMID) guidelines [1–3] recommend combining two tests in an algorithm to optimize the diagnosis. Despite its poor sensitivity when compared to toxigenic culture (60–81%) [4], toxin enzyme immunoassay (EIA) remains the preferred method for primary diagnosis in most laboratories because of its low cost and short turnaround time. In 2014, the results of a European multicenter prospective point-prevalence study of CDI in hospitalized patients with diarrhea highlighted the poor performances of the tests used in clinical laboratories [5], these poor performances being responsible for 25% of misdiagnoses.

To overcome this problem, nucleic acid amplification tests (NAATs) have been developed and are increasingly adopted. They are reported to have sensitivities ranging from 84 to 94% and short turnaround times compared with toxigenic culture [1]. Changing from EIA to more sensitive molecular tests is reported to increase the CDI detection during the transition year, with an increase up to 57% [4, 6–12]. The increased sensitivity of molecular tests has improved the laboratory detection of CDI cases, and current guidelines recommend their use in diagnosis as the first or second step [3]. However, a molecular test is difficult to implement in clinical laboratories because of its costs, the potential for confusion by physicians and overuse because of its novelty. It also cannot differentiate a truly infected patient from a colonized one [6, 13]. Its diagnostic accuracy is also variable and depends on CDI prevalence [14].

To increase the diagnostic accuracy of CDI, European guidelines have reinforced the importance of respecting strict conditions for performing tests on stool samples [2, 3]. Unformed stool samples from patients aged 3 years or older should be tested, and formed stool samples must be rejected [3]. Tests should be performed after at least 3 days in the hospital or after antibiotic treatment [2]. They should not have to be repeated within 7 days if they were positive and no test of cure is needed [15–17]. CDI testing should not be limited to samples with a specific physician’s request. Davies et al. [5] highlighted that over a third of laboratories detect *C. difficile* only upon a physician’s request. Dubberke et al. [18] showed that this request was inadequate, with 36% of the requests for non-diarrheal stools and 19% after laxative administration. Selection of the stool samples at the clinical laboratory level should improve the accuracy of the CDI diagnosis. However, it is unclear how closely the clinical laboratories follow these guidelines and whether they have an adequate stool sample rejection policy. The rate of rejected stool samples was reported to increase after NAAT implementation [6].

The purpose of this study was to estimate whether the implementation of NAATs resulted in changes in stool rejection policies between clinical laboratories that introduced NAATs and those that did not, between 2013 and 2014, in southwestern France. The stool rejection criteria and impact on the CDI positivity rate were determined. The correlation between a positive molecular test and a real case of CDI was also evaluated.

## Methods

A multicenter comparative retrospective survey was conducted in October 2014 in 12 clinical laboratories from hospitals in southwestern France. The laboratories provided hospital institutional data (size and type of institution) and details of current CDI laboratory diagnostic methods.

We compared the stool rejection policy of laboratories that introduced NAATs to that of laboratories that did not, between June 1 to September 30, 2013 and June 1 to September 30, 2014. A laboratory that introduced NAATs was defined as a laboratory using a novel molecular test in the first, second or third line of a diagnostic algorithm since October 2013. A laboratory without NAATs was defined as a laboratory using a non-molecular test such as EIA for toxins, EIA for glutamate dehydrogenase (GDH) and/or culture with toxin detection, and EIA detection of strains, alone or combined into algorithms.

The submitted survey included routine tests, the number of stools collected, the number of stools tested, the number of CDI-positive results, the number of tested patients and the stool rejection policy during the two periods. A positive result was defined as a positive test for free toxins or toxin genes. The positivity rate was defined as the ratio of positive stool samples to the total number of tested stools. The stool rejection rate was defined as the ratio of the number of tested stools to the total number of stool samples received at the laboratories. The repetition of the tests was evaluated by the number of tests performed per patient. The request criteria for performing tests were the following: only upon physician’s request, on all stool samples, on all diarrheal stool samples, on formed stool samples, for a patient 3 years or younger, for all patients 65 years or older, for patients hospitalized for a minimum of 3 days, on stool samples collected for 48 h or more, after a first positive sample during the same diarrheal episode, after a first negative sample during the same diarrheal episode, after a first positive sample within 7 days and for a test of cure.

The Chi square statistical test was used to assess differences.

To differentiate patients with CDI from asymptomatic carriers, we assessed whether the clinical picture of patients with a positive toxin-gene result met the criteria of CDI defined by Debast et al. [19]: significant diarrhea, ileus, toxic megacolon or pseudomembranous colitis.

## Results

Ten of the 12 requested laboratories answered the survey. Data from one laboratory were not included because of the introduction of a molecular test before 2013. The clinical laboratories included were from eight medium size [100,000–500,000 patient bed days (pbds)] and one large size (> 500,000 pbds) hospitals. Between June 1 and September 30, 2013, none of the laboratories from these nine hospitals performed molecular tests for CDI diagnosis (Table 1), instead they used a one- to three-step algorithm including GDH EIA, toxin EIA and toxigenic culture with toxin EIA detection of strains. After October 2013, four of the nine laboratories introduced NAAT in a two-step algorithm consisting of a screening test by GDH EIA (ImmunoCard^®^ *C. difficile* GDH, Meridian, Cincinnati, OH, USA) followed by a NAAT (*illumi*gene^®^, Meridian) for confirmation. A significant increase in the stool sample rejection rate was observed (Table 1) from 7.1 in 2013 to 11.3% in 2014 (p < 0.001). In contrast, in the five laboratories that did not adopt NAATs, the overall rate of rejected stool samples decreased from 11.7% in 2013 to 6.1% in 2014 (p < 0.001). Among these five laboratories, one observed a significant increase in the rejection rate (E), two others did not reject any samples in both periods (F and H), and two noted a decrease in the rejection rate (G and I). Among the laboratories that introduced NAATs, the rate of CDI-positive results was not significantly different (p = 0.07), except for one laboratory (B) (Table 1) that performed the largest number of tests (p = 0.003). In this laboratory, the positivity rate increased from 4.1 to 8.1%. No significant difference in the percentage of positive results was found in laboratories that did not introduce NAATs. Overall, there was no change in the mean number of stool samples tested globally or per patient (Additional file 1: Table S1) in the two groups of laboratories.

**Table 1:** Change in the number of stool specimens tested for *C. difficile* and the rate of positive specimens according to the laboratories between 2013 and 2014

Laboratory	Testing algorithm		Number of rejected samples			Number of positive results		
	2013	2014	2013	2014	p	2013	2014	p
A	GDH + EIA^a^	GDH + NAAT^d^	10/133 (7.5)	34/137 (24.8)	< 0.001	9/123 (7.3)	10/103 (9.7)	0.52
B	GDH + EIA^b^	GDH + NAAT^d^	89/776 (11.5)	83/653 (12.7)	0.47	29/687 (4.1)	46/570 (8.1)	0.003
C	GDH + EIA^b^	GDH + NAAT^d^	0/244 (0.0)	30/314 (9.6)	< 0.001	21/244 (8.6)	20/284 (7.0)	0.50
D	GDH + EIA^b^ + TC	GDH + NAAT^d^	0/225 (0.0)	0/193 (0.0)	*	18/225 (8.0)	14/193 (7.3)	0.77
Total			99/1378 (7.1)	147/1297 (11.3)	< 0.001	77/1279 (6.0)	90/1150 (7.8)	0.07
E	EIA^c^	GDH + EIA^a^	6/42 (14.2)	13/44 (29.5)	0.88	2/42 (4.7)	6/31 (19.4)	0.09
F	GDH + EIA^b^	GDH + EIA^b^	0/73 (0.0)	0/85 (0.0)	*	6/73 (8.2)	4/85 (4.7)	0.37
G	GDH + EIA^b^	GDH + EIA^b^	47/178 (26.4)	25/152 (16.4)	0.82	20/147 (13.6)	17/127 (13.4)	0.96
H	GDH + EIA^b^ + TC	GDH + EIA^b^ + TC	0/228 (0.0)	0/267 (0.0)	*	12/228 (5.3)	8/267 (3.0)	0.20
I	GDH + EIA^b^ + TC	GDH + EIA^b^ + TC	23/211 (10.9)	5/162 (3.1)	0.008	17/188 (9.0)	11/157 (7.0)	0.49
Total			76/648 (11.7)	43/710 (6.1)	< 0.001	57/678 (8.4)	46/667 (6.9)	0.30

The number of rejected samples is expressed as the number of samples rejected/total number of samples collected (percent). The number of *C.* *difficille* infection (CDI)-positive samples is expressed as the number of CDI-positive samples/total number of samples tested (percent). The number of tests per patient is expressed as the number of tested samples/number of patients (ratio). *A to D*, laboratories that introduced nucleic acid amplification tests (NAATs). *E to I*, laboratories that did not introduce NAATs

*GDH* glutamate dehydrogenase by enzyme immunoassay, *EIA* toxin enzyme immunoassay, *TC* toxigenic culture

Statistical analysis was performed using the Chi square test. A p value < 0.05 was considered significant

*Not applicable

Methods: ^a^*C. Diff*. Quik Chek Complete©, Alere (Waltham, MA, USA); ^b^*C. Diff*. Quik Chek GDH©, Alere and TOX A/B Quik Chek©, Alere; ^c^. And TOX A/B Quik Chek©, Alere; ^d^ImmunoCard^®^ *C. difficile* GDH, Meridian (Cincinnati, OH, USA) and NAAT *illumi*gene^®^, Mridiane

In 2014, the laboratories that introduced NAATs were no longer limiting their *C.* *difficile* detection only to physicians’ requests; they performed tests on stools from all patients hospitalized for 3 days with diarrhea and no longer tested the stool samples of patients with a positive CDI result obtained within 7 days (Additional file 2: Table S2). The B laboratory was also the only one to test all diarrheal stool samples collected. The laboratories that did not implement NAATs did not adopt these criteria in 2014, and two of them performed tests on all collected samples. There was no change in their stool rejection policy between the two periods.

The correlation between positive NAATs and the clinical picture was evaluated. Seventy of 73 patients (95.8%) who had a positive NAAT result had a clinical picture consistent with CDI in terms of the ESCMID criteria (Table 2).

**Table 2:** Clinical correlation of the positive nucleic acid amplification test (NAAT) results in 2014 in the 4 laboratories that introduced NAATs as a second- or third-line test

Laboratory	Number of positive NAAT results	Number of patients	Chart review in favor of CDI
A	10	9	9 (100.0%)
B	46	41	39 (95.1%)
C	20	15	14 (93.3%)
D	14	8	8 (100.0%)
Total	90	73	70 (95.8%)

The chart review was based on the clinical definition of *C.* *difficile* infection in the ESCMID recommendations [1]

## Discussion

The clinical laboratories that introduced NAATs showed a significantly increase in the rate of rejected stool samples. Previously, Cohen et al. [6] reported that laboratories adopted a more stringent stool rejection policy after NAAT implementation in the USA. These authors did not specifically ask laboratories to explain the rationale for adopting more stringent policies, and no studies were conducted to evaluate the stool rejection criteria adopted in European laboratories after implementation of a new testing algorithm for CDI diagnosis. In our study, three criteria based on the current diagnosis recommendations [2] were adopted by the NAAT laboratories: they no longer limited their *C.* *difficile* detection to physicians’ requests, they performed tests on patients hospitalized for a minimum of 3 days with diarrhea and they no longer tested the stool samples of patients with a positive CDI result obtained within 7 days. The possibility for the microbiology laboratory to cancel repeat tests within 1 week is thought to have contributed to a significant decrease in the number of tested stools [7]. A reduction in the number of tested samples from 48 to 38.2% was previously reported after NAAT implementation [6, 7, 20]. This reduction was not observed in our study. The number of stool samples tested per patient was stable, highlighting that the laboratories controlled the number of tests and limited the overuse of NAATs (Additional file 1: Table S1). The cancellation of repeated tests should be considered as a priority by clinical laboratories, but we cannot conclude that only one criterion is needed for effective action. The laboratories must adopt all of the stool rejection recommendations [1–3] to improve CDI diagnosis.

The use of suboptimal clinical tests for CDI diagnosis is still prevalent throughout Europe [5, 21, 22]. Both the IDSA [1] and ESCMID [2, 3] suggest a GDH EIA-based two-step algorithm or use of NAAT alone to improve diagnostic accuracy. The French guidelines at the time, did not include these diagnosis algorithms [23], which were added to the revised guidelines in 2015. Only one laboratory used toxin EIA alone in 2013, while the others used two- or three-step algorithms. In 2014, all of the laboratories (100%) chose a recommended two- or three-step algorithm [2] with an initial screening by GDH EIA, which is superior to the 65 and 85% reported in France and the UK, respectively, in 2015 [24]. The tests were all performed the day the stool samples were received by the laboratories. The current guidelines for CDI laboratory diagnosis [2] were adopted in southwestern France. PCR alone was described to be more sensitive than the GDH EIA-based algorithm, but the data are conflicting [4, 25, 26]; laboratories chose the two-step algorithm because of its lower cost per patient. Although the laboratories that did not implement NAATs used the recommended testing algorithm, they did not follow the recommendations carefully and had a poor stool rejection policy. Greater efforts to improve stool rejection policies are necessary.

The rate of positive results increased significantly for only one laboratory that introduced NAATs and performed the largest number of tests. Previous studies described an increase in CDI-positive results of up to 57% with PCR testing alone [7, 8, 10–12, 20], and this increase was expected for all laboratories that implemented NAATs but was only observed for laboratory B (Table 1). In Europe, the mean annual CDI testing and CDI-positive rates were reported to be significantly higher in medium-sized hospitals (46.2/10,000 and 3.3/10,000 pbds, respectively) compared to large hospitals (28.6/10,000 and 1.5/10,000 pbds, respectively) [24]. The incidence of CDI in France ranges from 2.9 to 3.6 cases/10,000 pbds [24, 27]. In contrast, Cohen et al. did not observe a systematic increase in positivity rates after the implementation of a multistep algorithm involving NAATs [6]. The enrollment of patients, the low level of awareness of physicians and the seasonal pattern of the incidence of CDI could explain our results in the medium-sized hospitals [28–31]. The relatively low number of specimens processed by these laboratories and our short observation period could also explain these results. Regarding the positivity rate, the study has several limitations: this is a nonrandomized and retrospective study that included a network of different sized hospitals.

Concerns about the detection of colonized patients using NAAT have been raised and emphasize the importance of testing patients with clinically significant diarrhea in order to avoid false-positive tests. We assessed whether the clinical picture of patients with a positive toxin-gene result met the criteria of CDI defined by Debast et al. [19]. Planche et al. [13] suggested that the presence of free toxins could best define true or severe CDI. The presence of free toxins was significantly associated with unfavorable CDI, with an increase in white blood cells and a higher mortality rate. Using the clinical criteria defined by the European recommendations [9], there was a strong correlation between CDI-positive NAAT results and the clinical picture. The Meridian algorithm (EIA ImmunoCard^®^ *C. difficile* GDH, followed by NAAT *illumi*gene^®^ for toxin gene), along with an adequate stool rejection policy, provided a true CDI diagnosis.

## Conclusion

Clinical laboratories will continue to adopt NAATs as part of their routine testing methods due to the higher sensitivity and short turnaround time of these tests. This is the first report on the impact of the implementation of NAATs on stool rejection policies in Europe. The clinical laboratories took advantage of the change in the testing algorithm including NAATs to adopt the current recommendations. The current European guidelines have to be followed combined with by a correct stool rejection policy. The adoption of the largest number of the recommended criteria is necessary to have an effective rejection policy. An increase in the rate of rejected stool samples was observed because hospitals no longer limited their *C.* *difficile* detection upon physicians’ requests only and furthermore they limited repeat tests. NAAT implementation will likely improve compliance with recommended stool rejection policies and improve detection of *C.* *difficile*.

## Additional files

**Additional file 1: Table S1.** Change in the number of stool specimens tested per patient expressed as a ratio of the number of tested samples/number of patients. Statistical analysis was performed using the Chi square test. p value <0.05 was considered significant. * Not applicable.

**Additional file 2: Table S2.** Change in the stool rejection policy according to the laboratories between 2013 and 2014. Data are the number of laboratories that adopted the requested criteria for performing tests among the four laboratories that introduced nucleic acid amplification tests (NAATs) and the five that did not introduce NAATs, between the studied periods.
