# Supplementary material for: Impact of the introduction of a nucleic acid amplification test for Clostridium difficile diagnosis on stool rejection policies
Source: Gut Pathog. 2018 May 30;10:19. doi: 10.1186/s13099-018-0245-x (PMC5975266; doi:10.1186/s13099-018-0245-x)
Supplement: Supplementary file 1 — Additional file 1: Table S1. Change in the number of stool specimens tested per patient expressed as a ratio of the number of tested samples/number of patients. Statistical analysis was performed using the Chi square test. p value <0.05 was considered significant. * Not applicable. [file 13099_2018_245_MOESM1_ESM.docx]

**Table S1. Change in the number of stool specimens tested** per patient expressed as a ratio of the number of tested samples/number of patients. Statistical analysis was performed using the Chi square test. p value <0.05 was considered significant. * not applicable.

|  | **2013** |  |  | **2014** | |  | |  | |
| --- | --- | --- | --- | --- | --- | --- | --- | --- | --- |
| **Laboratory** | **Number of tested specimens** | **Number of patients** | **Ratio** | **Number of tested specimens** | **Number of patients** | | **Ratio** | | **p value** |
| **A** | 123 | 101 | 1.2 | 103 | 75 | | 1.4 | | 0.553 |
| B | 687 | 538 | 1.3 | 570 | 509 | | 1.1 | | 0.117 |
| C | 244 | 180 | 1.4 | 284 | 208 | | 1.4 | | 0.957 |
| D | 225 | 176 | 1.3 | 193 | 154 | | 1.3 | | 0.893 |
| **Total** | **1279** | 995 | **1.3** | **1150** | **946** | | **1.2** | | **0.360** |
| E | 42 | 36 | 1.2 | 31 | 28 | | 1.1 | | 0.880 |
| F | 73 | 73 | 1.0 | 85 | 83 | | 1.0 | | 0.916 |
| G | 147 | 121 | 1.2 | 127 | 113 | | 1.1 | | 0.662 |
| H | Incomplete data | Incomplete data | * | Incomplete data | Incomplete data | | * | | * |
| I | 188 | 157 | 1.2 | 157 | 136 | | 1.2 | | 0.819 |
| **Total** | **450** | I | **1.2** | **400** | **360** | | **1.1** | | **0.651** |
