# Supplementary material for: Impact of the introduction of a nucleic acid amplification test for Clostridium difficile diagnosis on stool rejection policies
Source: Gut Pathog. 2018 May 30;10:19. doi: 10.1186/s13099-018-0245-x (PMC5975266; doi:10.1186/s13099-018-0245-x)
Supplement: Supplementary file 2 — Additional file 2: Table S2. Change in the stool rejection policy according to the laboratories between 2013 and 2014. Data are the number of laboratories that adopted the requested criteria for performing tests among the four laboratories that introduced nucleic acid amplification tests (NAATs) and the five that did not introduce NAATs, between the studied periods. [file 13099_2018_245_MOESM2_ESM.docx]

**Table S2. Change in the stool rejection policy according to the laboratories between 2013 and 2014.** Data are the number of laboratories that adopted the requested criteria for performing tests among the four laboratories that introduced nucleic acid amplification tests (NAATs) and the five that did not introduce NAATs, between the studied periods.

| Requested criteria for performing tests | Laboratories that introduced NAATs (4) | | Laboratories that did not introduce NAATs (5) | |
| --- | --- | --- | --- | --- |
|  | 2013 | 2014 | 2013 | 2014 |
| Only upon physician’s request | 4/4 | 1/4 | 2/5 | 2/5 |
| On all stool samples | 0/4 | 0/4 | 2/5 | 2/5 |
| On all diarrheal stool samples | 1/4 | 1/4 | 3/5 | 3/5 |
| On formed stool samples | 0/4 | 0/4 | 2/5 | 2/5 |
| For patients three years or younger | 0/4 | 0/4 | 0/5 | 0/5 |
| For all patients 65 years or older | 0/4 | 0/4 | 0/5 | 0/5 |
| For patients hospitalized for a minimum of three days | 1/4 | 4/4 | 2/5 | 2/5 |
| On stool samples collected for 48 h or more | 1/4 | 3/4 | 1/5 | 1/5 |
| After a first positive sample during the same diarrheal episode | 3/4 | 0/4 | 3/5 | 3/5 |
| After a first negative sample during the same diarrheal episode | 3/4 | 3/4 | 3/5 | 3/5 |
| After a first positive sample within seven days and for a test of cure | 3/4 | 0/4 | 3/5 | 3/5 |
